# Supplementary material for: Single-Cell Profiling Reveals Immune-Based Mechanisms Underlying Tumor Radiosensitization by a Novel Mn Porphyrin Clinical Candidate, MnTnBuOE-2-PyP5+ (BMX-001)
Source: Antioxidants (Basel). 2024 Apr 17;13(4):477. doi: 10.3390/antiox13040477 (PMC11047573; doi:10.3390/antiox13040477)
Supplement: Supplementary file 1 [file antioxidants-13-00477-s001.zip › Supplementary Materials (revised, antioxidants-28822362).pdf]

Supplementary Materials for

Single-cell profiling reveals immune-based mechanisms underlying the tumor radiosensitization by a novel Mn porphyrin clinical candidate, $MnTnBuOE\text{-}2\text{-}PyP^{5+}$ (BMX-001)

Sun Up Noh[1,2,†], Jinyeong Lim[3,4,†], Sung-Won Shin[1], Yeeun Kim[1], Woong-Yang Park[3,4], Ines Batinic-Haberle[5], Changhoon Choi[1,*] and Won Park[1,2,*]

[1] Department of Radiation Oncology, Samsung Medical Center, Seoul 06351, Republic of Korea; chocolatebox919@gmail.com (S.U.N.); camuserik@gmail.com (S.-W.S.); yeeun17.kim@sbri.co.kr (Y.K.); chchoi93@gmail.com (C.C.); wonro.park@samsung.com (W.P.)

[2] Sungkyunkwan University School of Medicine, Seoul 06351, Republic of Korea

[3] Department of Health Sciences and Technology, Samsung Advanced Institute for Health Sciences and Technology, Sungkyunkwan University, Seoul 06351, Republic of Korea; aster1217@gmail.com (J.L.); woongyang.park@samsung.com (W.-Y.P.)

[4] Samsung Genome Institute, Samsung Medical Center, Sungkyunkwan University, Seoul 06351, Republic of Korea

[5] Department of Radiation Oncology, Duke University School of Medicine, Durham, NC 27710, USA; ibatibic@duke.edu (I.B.-H.)

* Correspondence: wonro.park@samsung.com (W.P.); chchoi93@gmail.com (C.C.) ; Tel.: +82-2-3410-2600 (W.P.)

† These authors contributed equally to this work

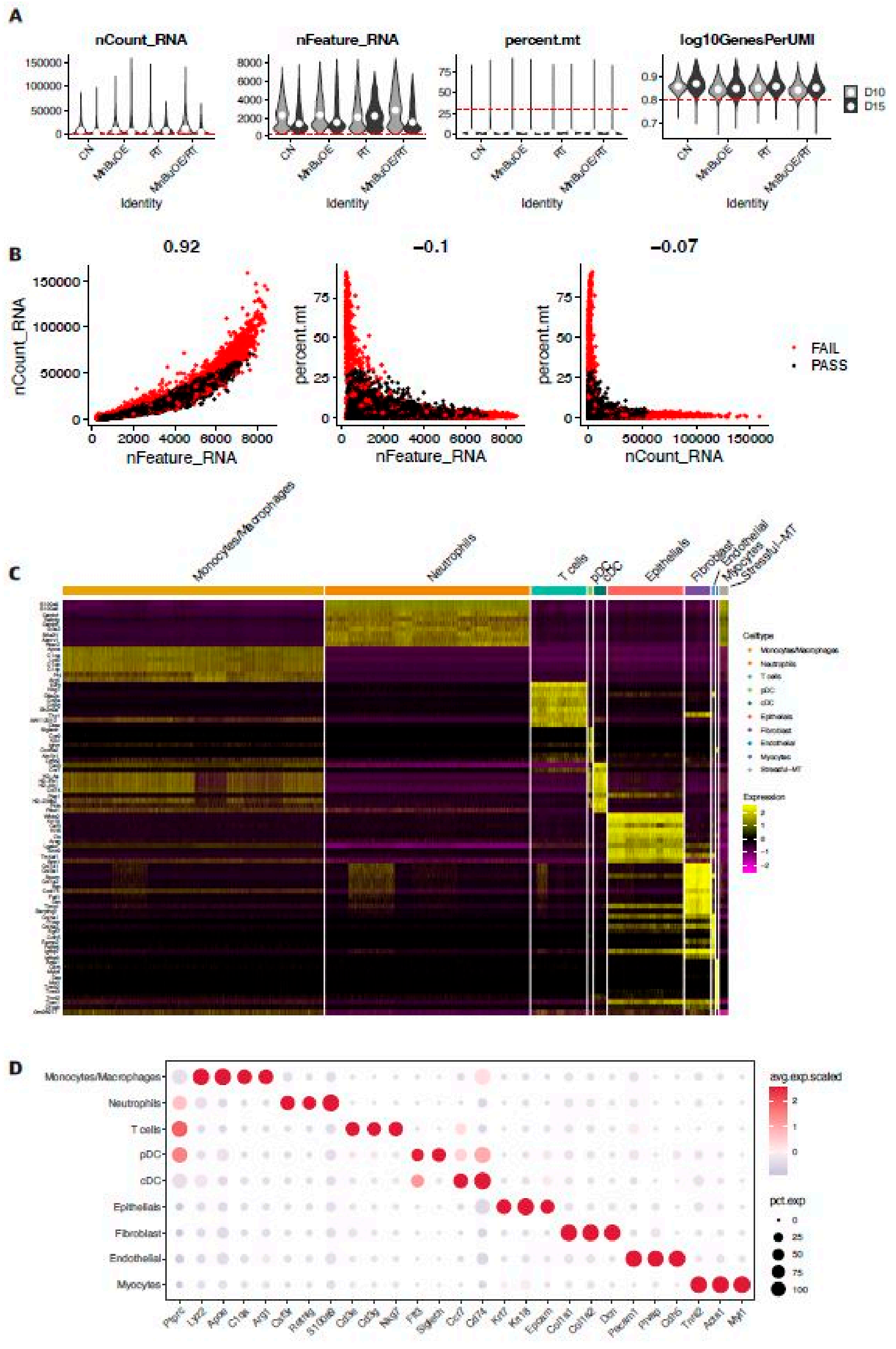

Figure S1. Quality-control of scRNA-seq and annotation of major clusters.

A. Violin plots visualizing the number of RNA count (nCount_RNA), the number of detected genes (nFeature_RNA), the percentage of expressing mitochondrial genes (percent.mt), and log scales of the number of genes per UMI (log10GenesPerUMI) for each experimental group and different time points, across distinct experimental groups and time points. The median values for each metric were denoted by white circles, and category thresholds were indicated by red dashed lines. **B.** A

scatterplot illustrates the relationship between nFeature_RNA, nCount_RNA, and percent.mt. Quality-passed cells were denoted by black dots, while cells not meeting quality control criteria were represented by red dots. **C.** Heatmap exhibiting distinct expression patterning of the top ten most variable genes for each cluster among the cell clusters. Gene expression levels from low to high are indicated by a color gradient from purple to yellow. **D.** A dot plot visualizes the expression features of selected signature genes for each cell type. The dots' colors, ranging from blue to red, represented gene expression levels from low to high. Dot size indicates the percentage of cells expressing specific genes. scRNA-seq, single-cell RNA sequencing; UMI, unique molecular identifier.

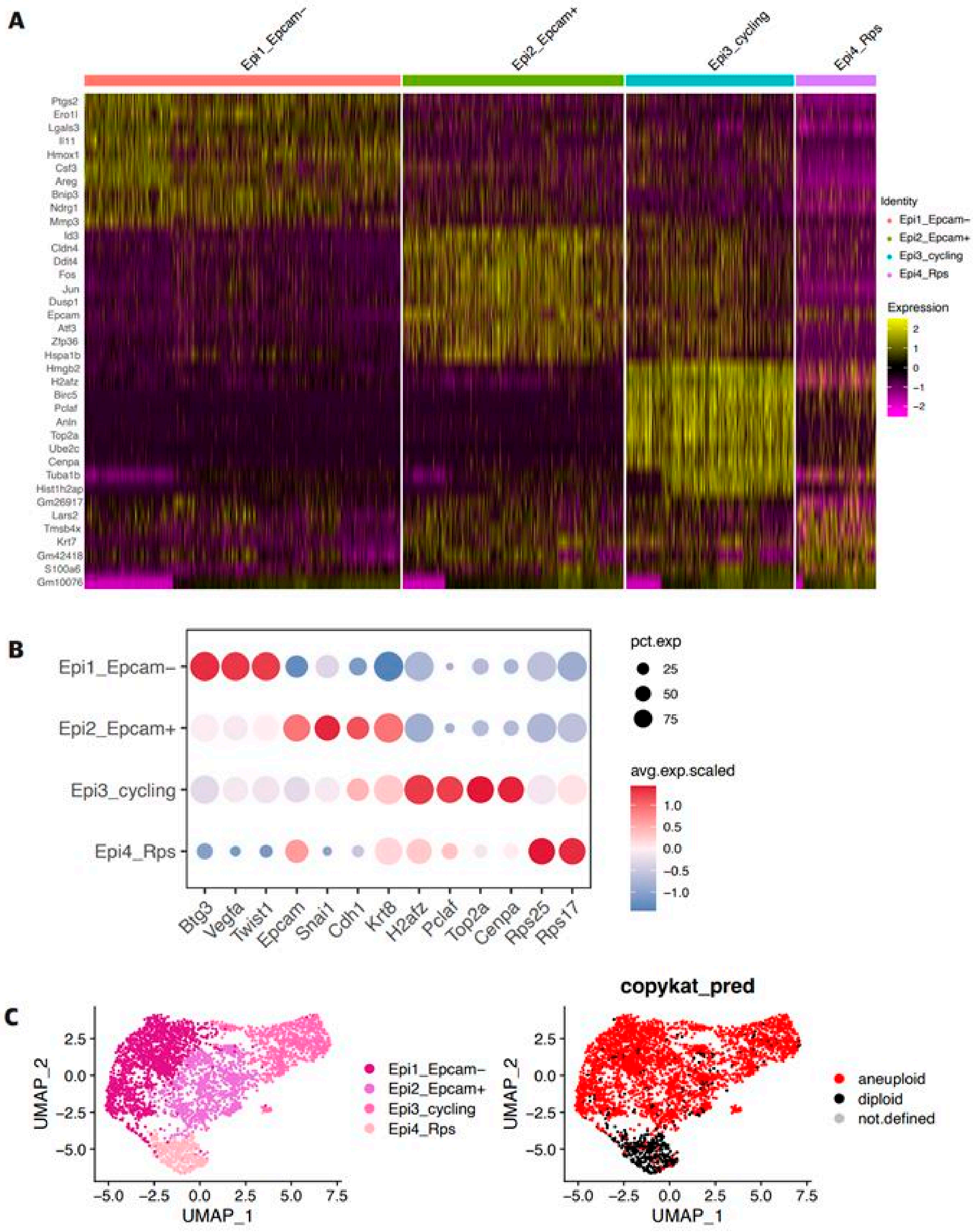

Figure S2. Identification of epithelial cell clusters.

A. The heatmap displays the unique expression patterns of the ten most variable genes within four epithelial cell clusters, showcasing distinct patterning among the clusters. Graduating colors from purple to yellow indicate gene expression levels ranging from low to high. **B.** A dot plot illustrates the expression characteristics of 13 epithelial genes for individual cell types. The dots' colors, spanning from blue to red, depicted gene expression levels varying from low to high. The size of each dot corresponds to the percentage of cells expressing the particular genes. C. UMAP plot

indicated epithelial cell subtypes (left), and distinguished cancer cells (aneuploid) and normal cells (diploid) within epithelial cells using CopyKAT analysis (right).

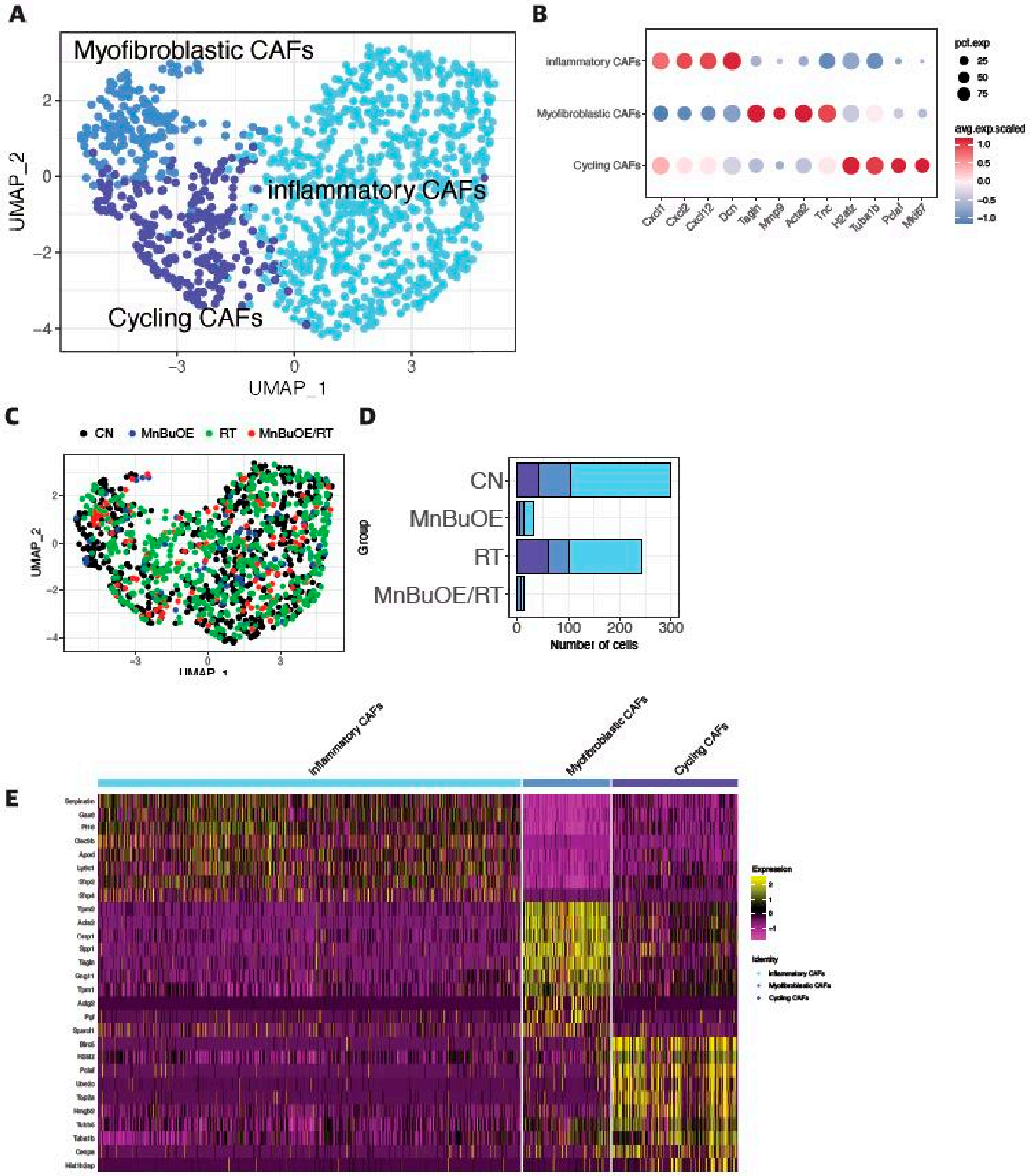

Figure S3. Decreased number of fibroblasts following treatment with MnBuOE and irradiation. **A.** UMAP plot of fibroblasts indicated three subtypes: inflammatory CAFs, myofibroblastic CAFs, and cycling CAFs (n=1,390). **B.** Violin plots showing expression of genes related to markers of each fibroblast subtype: inflammatory CAFs (Cxcl1, Cxcl2, and Cxcl12), myofibroblastic CAFs (Tagln, Mmp9, and Acta2), and cycling CAFs (H2afz, Tuba1b, and Mki67). **C, D.** Distribution of fibroblasts in each experimental group, color-coded by fibroblast subtypes. Most of the CAFs including inflammatory or myofibroblastic CAFs were reduced in the MnBuOE treatment groups (MnBuOE, MnBuOE only; MnBuOE/RT, MnBuOE and irradiation), compared to the CN (control) and RT (irradiation only) groups. **E.** Within the three clusters of CAFs, the heatmap reveals exclusive expression patterns of the ten most variable genes. The color spectrum, progressing from purple

to yellow, serves to illustrate gene expression levels, spanning from low to high. MnBuOE, MnTnBuOE-2-PyP^{5+}; UMAP, uniform manifold approximation and projection; CAFs, cancer-associated fibroblasts; MnBuOE/RT, MnTnBuOE-2-PyP^{5+}/radiation therapy.

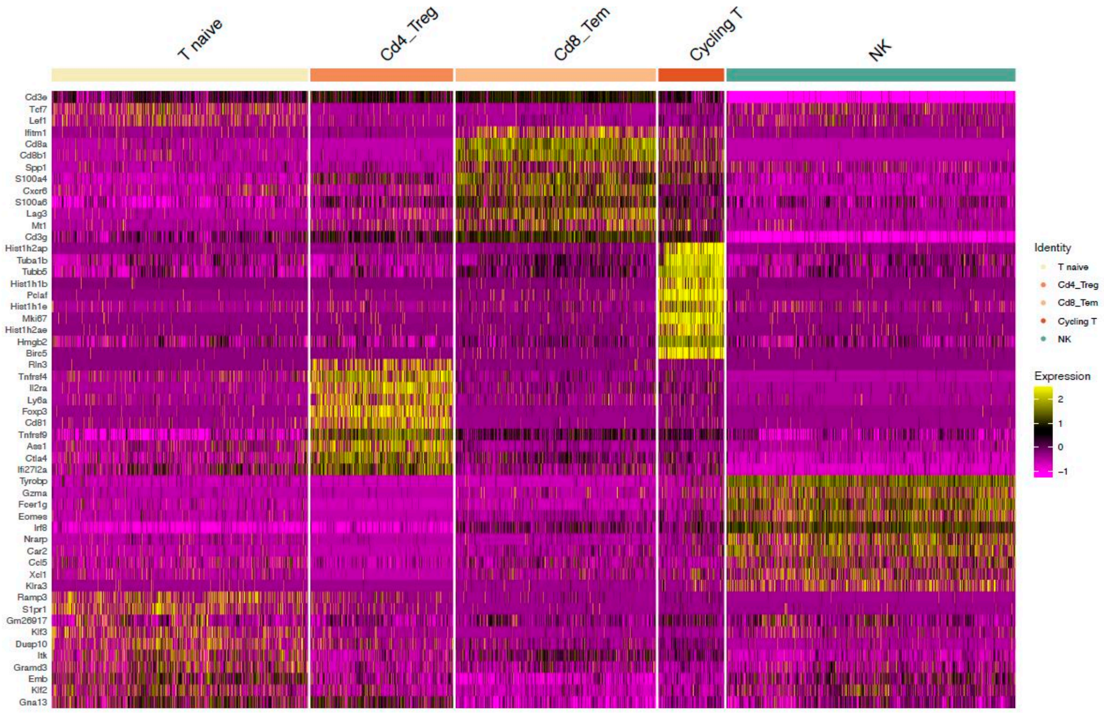

Figure S4. Characterizing of T and NK cell clusters.

The heatmap illustrates the distinct expression patterns of the most variable genes within four clusters of T cells and NK cells. A color gradient spanning from purple to yellow signifies gene expression levels, transitioning from low to high. NK, natural killer.

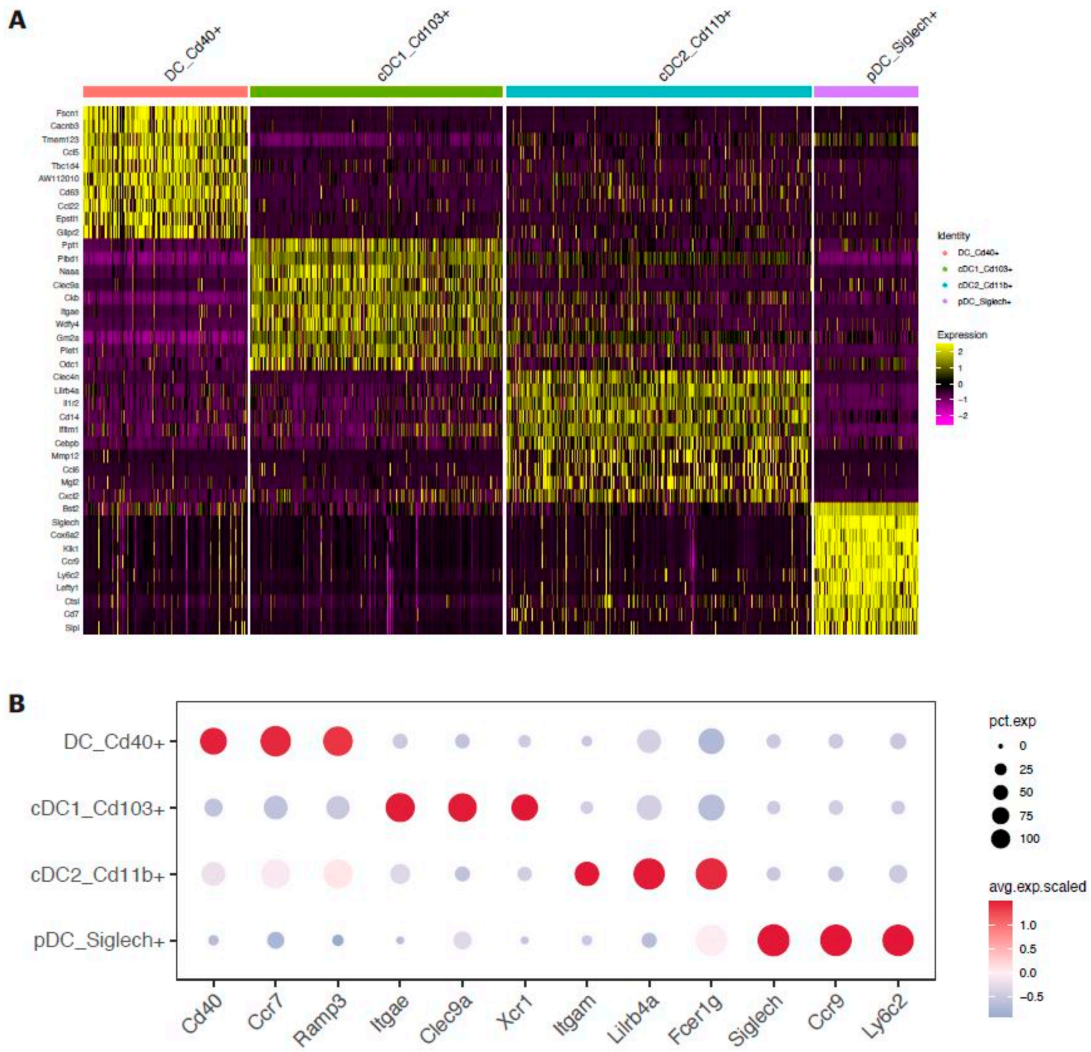

Figure S5. Identification of dendritic cell clusters.

A. Heatmap plots showing the expression of the top ten marker genes of four dendritic cell types identified in the analysis. **B.** A dot plot illustrates the expression characteristics of 12 marker genes for individual cell types.

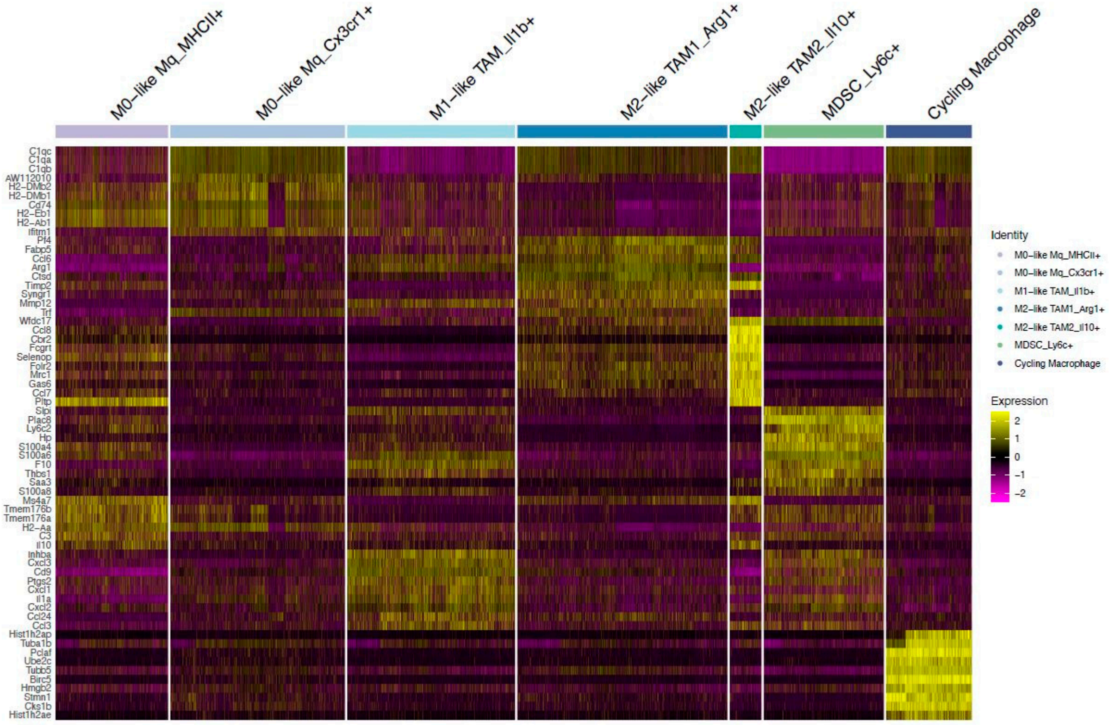

Figure S6. Characterization of seven myeloid cell subsets.

Heatmap depicts the expression patterns of the ten marker genes associated with seven distinct myeloid cell types identified.

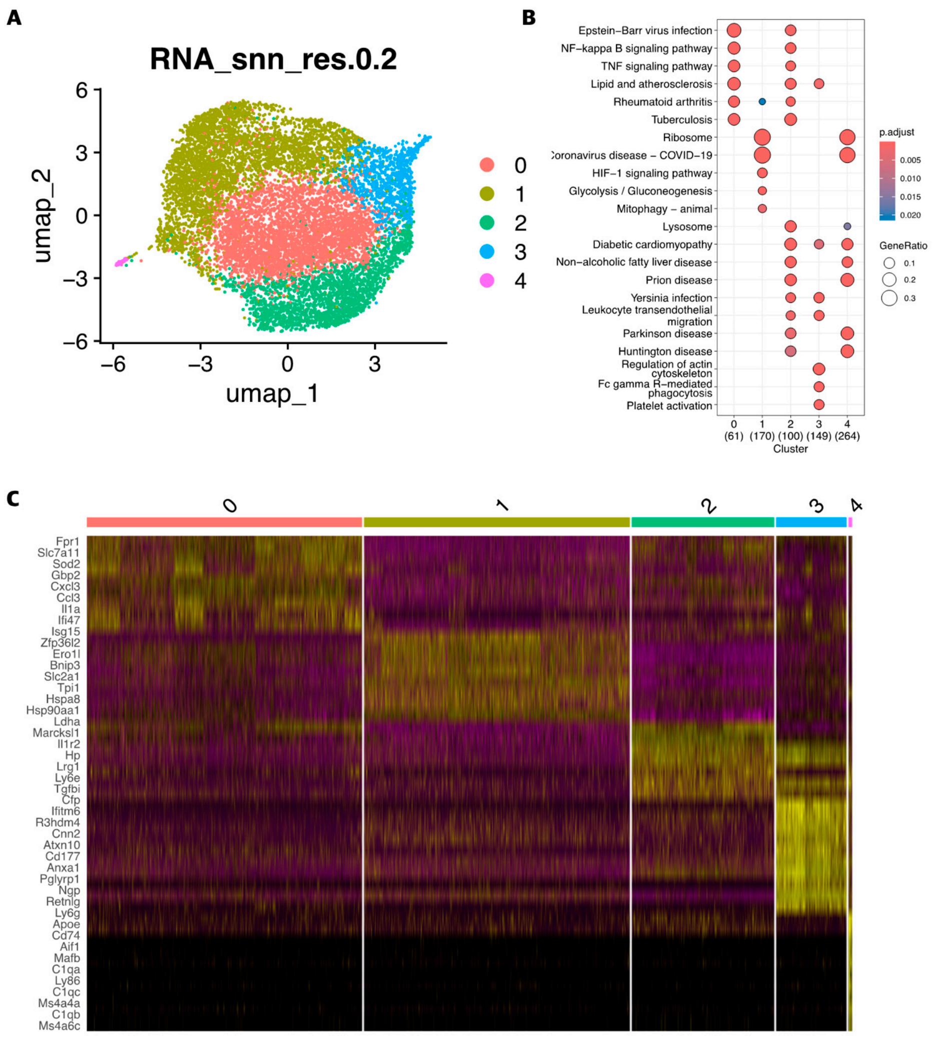

Figure S7. Identification of neutrophil clusters.

A. Dimplot of five neutrophil clusters. **B.** Functional pathways analysis using KEGG pathways based on the top marker genes within each cluster. **C.** Top expression genes within each neutrophil clusters.

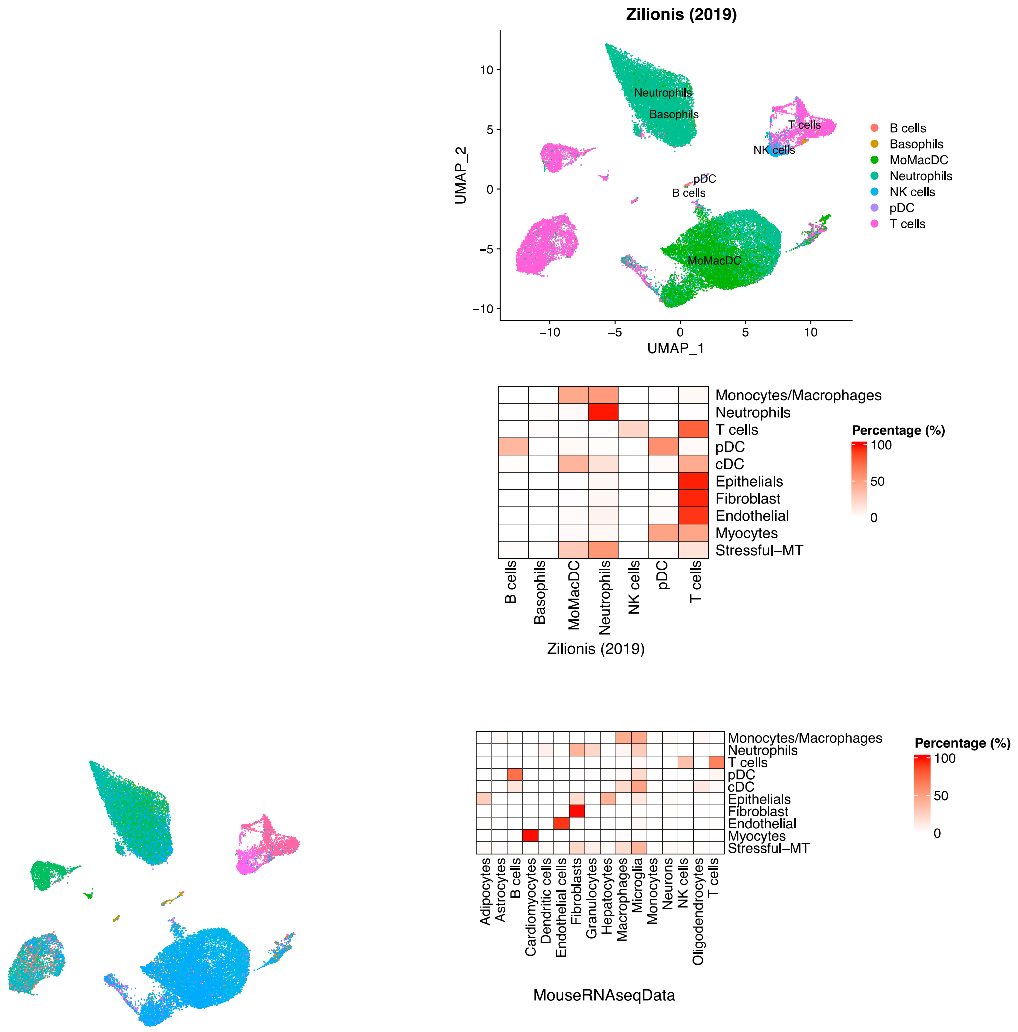

Figure S8. Validation of global annotations using multiple methods.

A. Predicted annotations of 19 cell types visualized in a dimensionality reduction plot, with corresponding percentages of matching rates against cluster-based annotations (top) and ImmGendata (Heng et al. 2008) obtained from the celldex R package (bottom). **B.** Utilization of scAnnotate to predict cell annotations into 7 immune cell types using references (zilionis et al. 2019), with a heatmap representation illustrating the percentage of matching rates compared to cluster-based annotations. **C.** Predicted annotations using mouseRNAseqData reference (Benayoun et al. 2019) in celldex via scAnnotate, accompanied by a corresponding heatmap showing the matching rates.
