# Supplementary figures and images for: Single-Cell Profiling Reveals Immune-Based Mechanisms Underlying Tumor Radiosensitization by a Novel Mn Porphyrin Clinical Candidate, MnTnBuOE-2-PyP5+ (BMX-001)
Source: Antioxidants (Basel). 2024 Apr 17;13(4):477. doi: 10.3390/antiox13040477 (PMC11047573; doi:10.3390/antiox13040477)

**A**

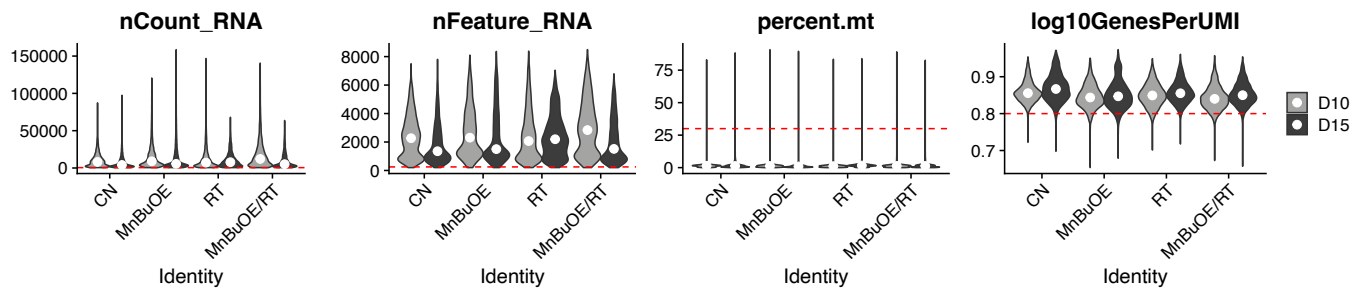**B**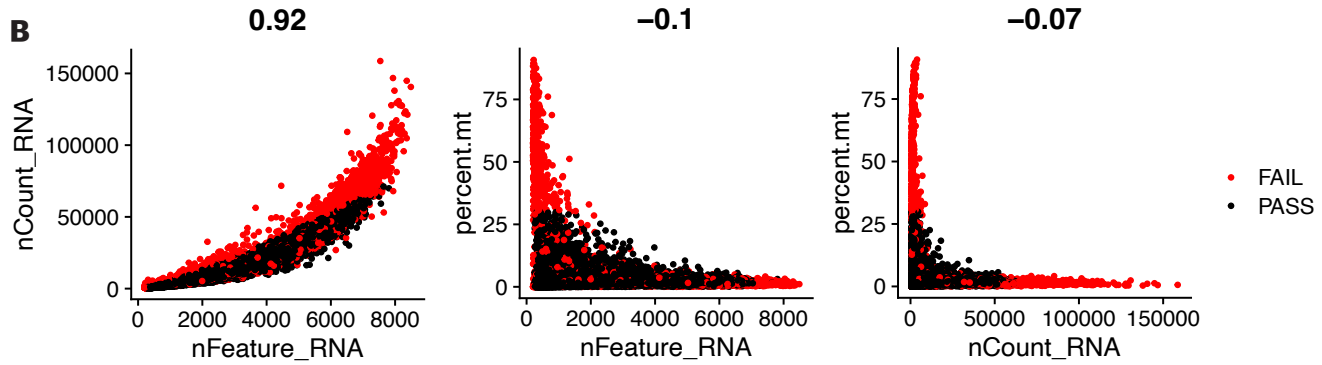

**C**

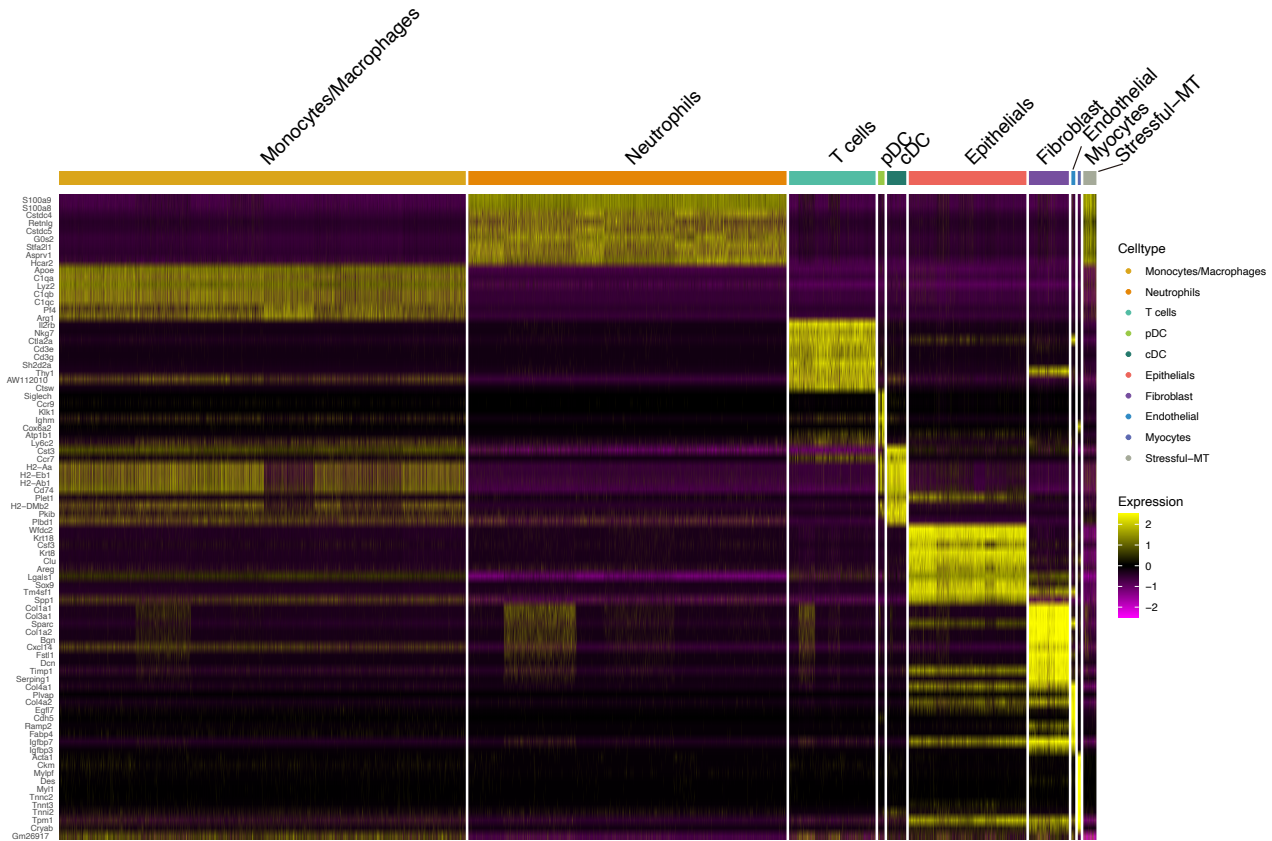

## D

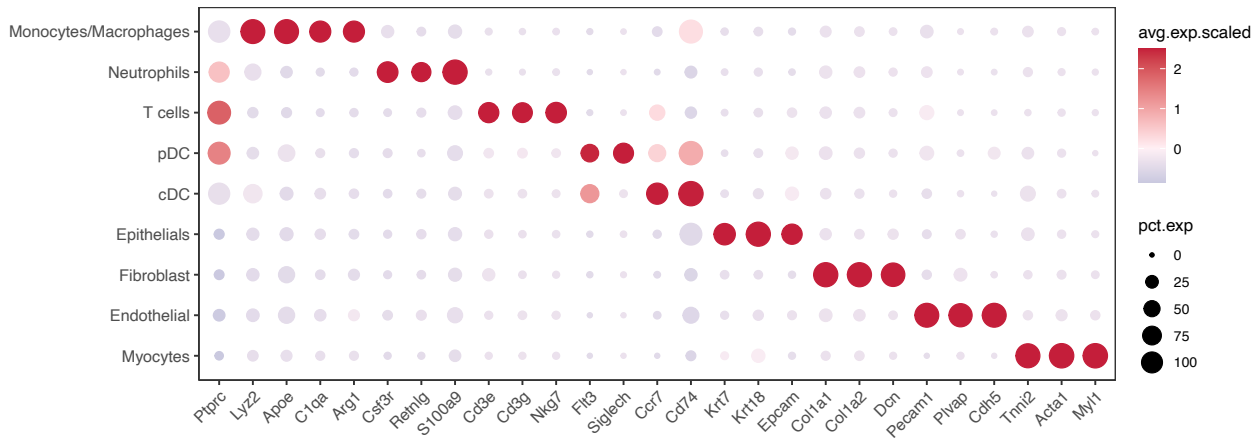

Supplement: Supplementary file 1 [file antioxidants-13-00477-s001.zip › Figure S1.pdf]

**A**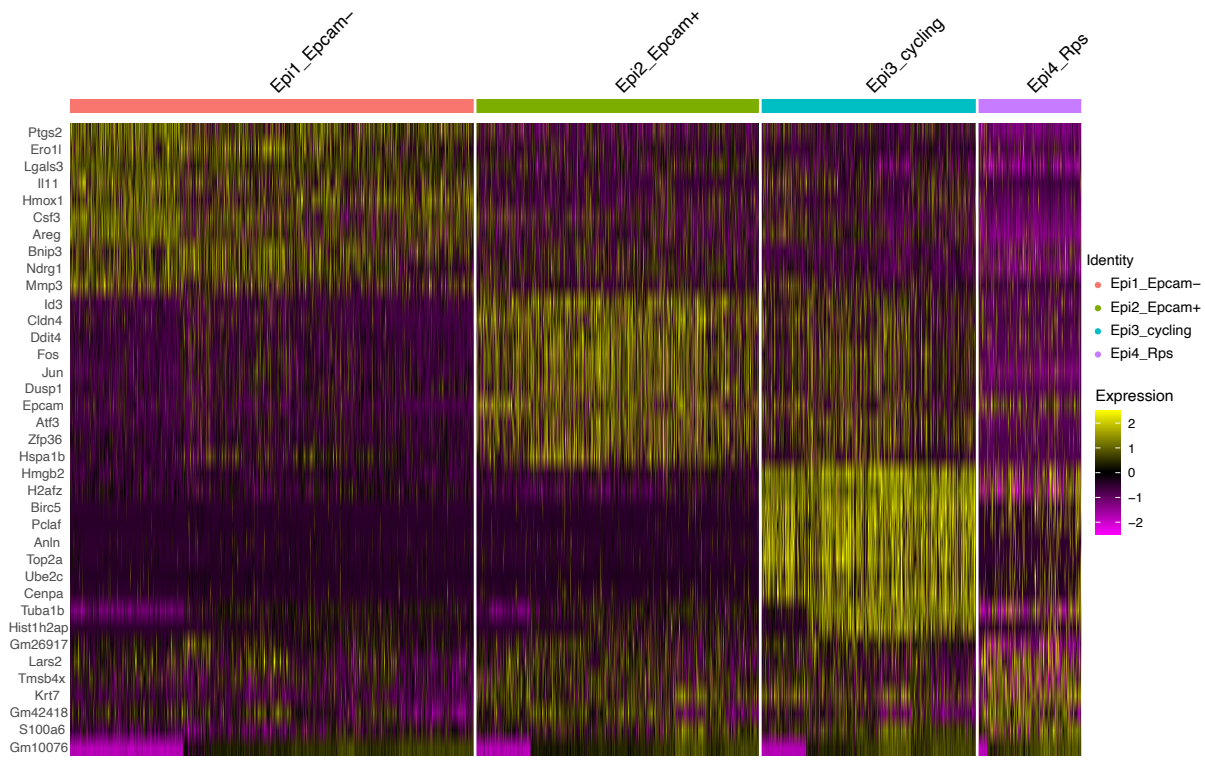**B**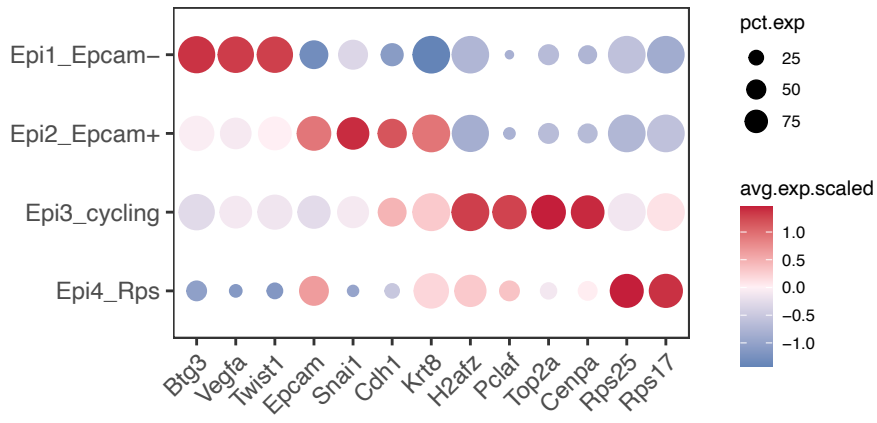**C**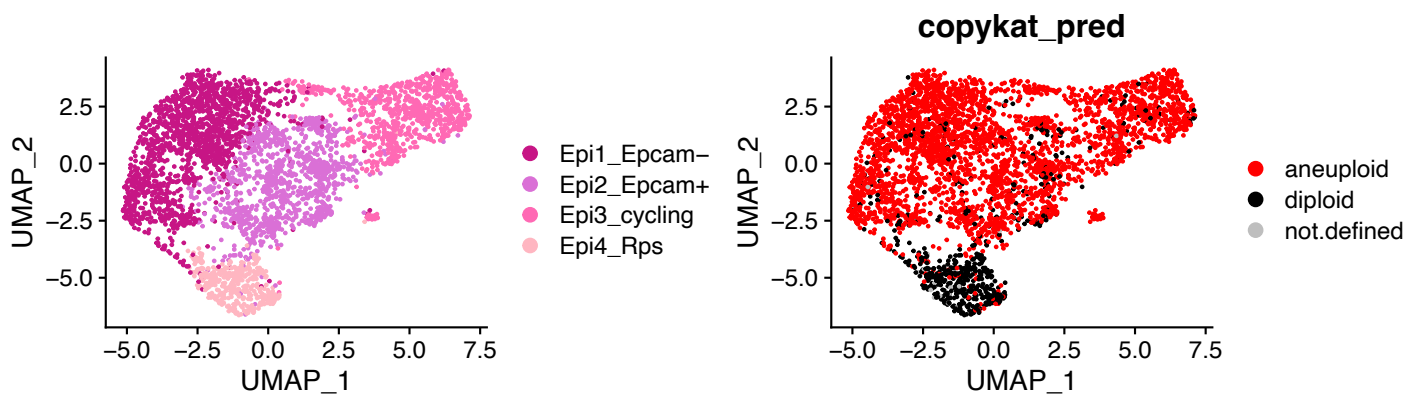

Supplement: Supplementary file 1 [file antioxidants-13-00477-s001.zip › Figure S2.pdf]

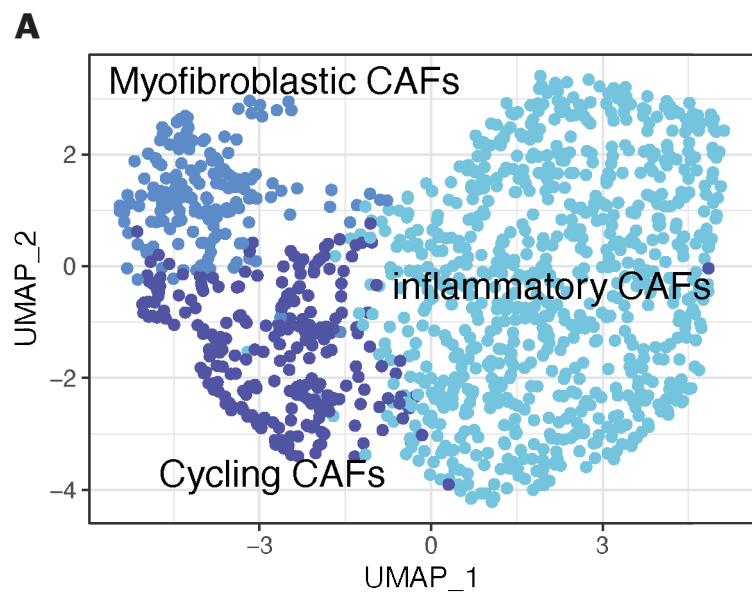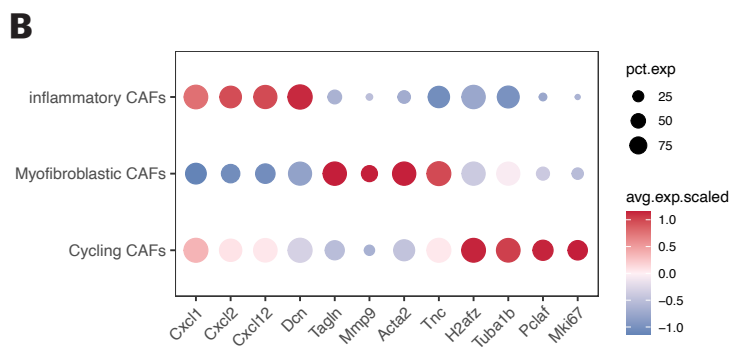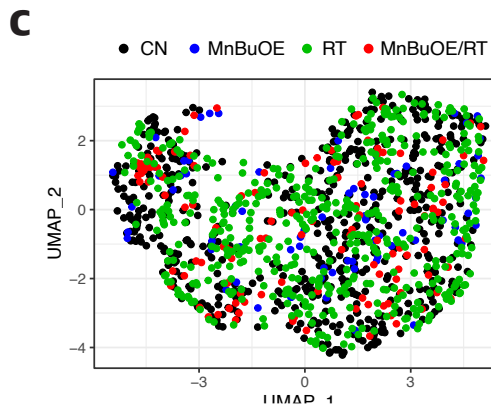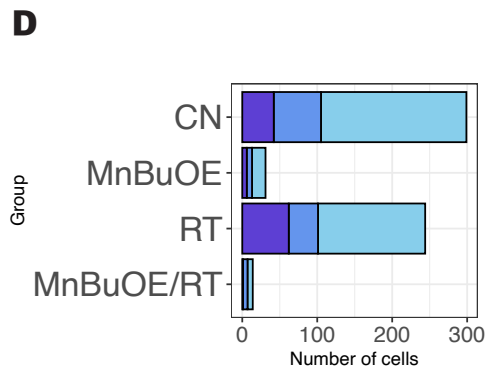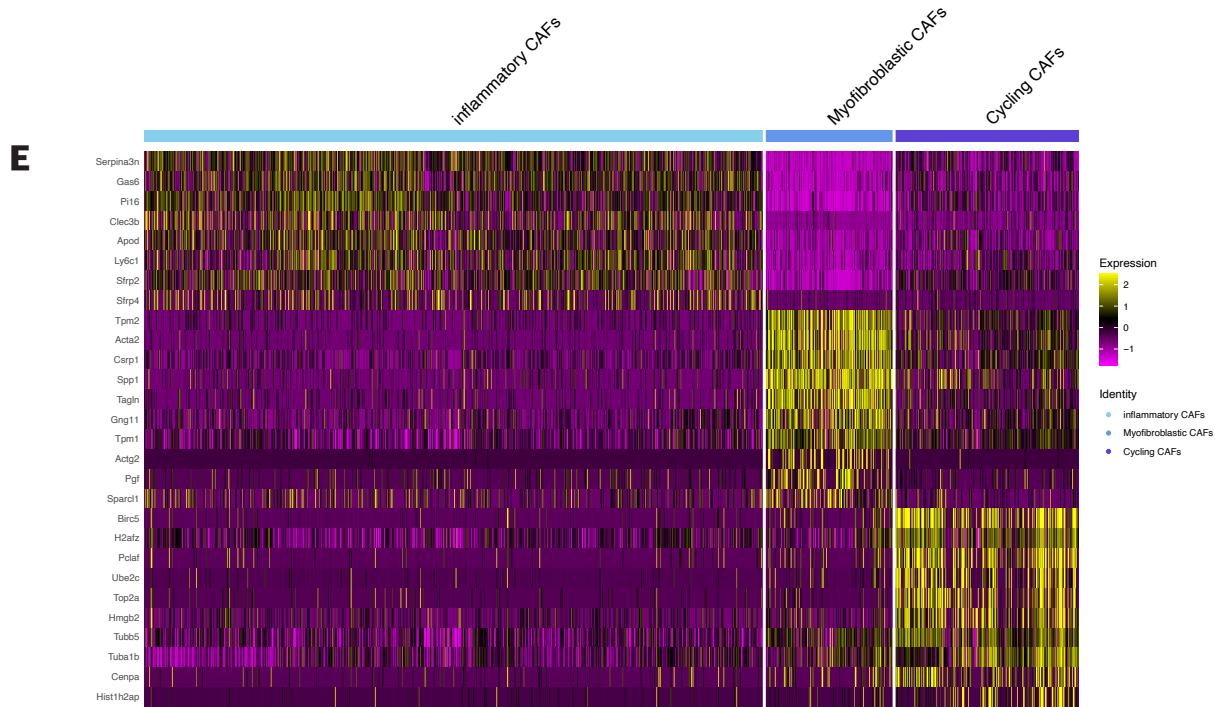

Supplement: Supplementary file 1 [file antioxidants-13-00477-s001.zip › Figure S3.pdf]

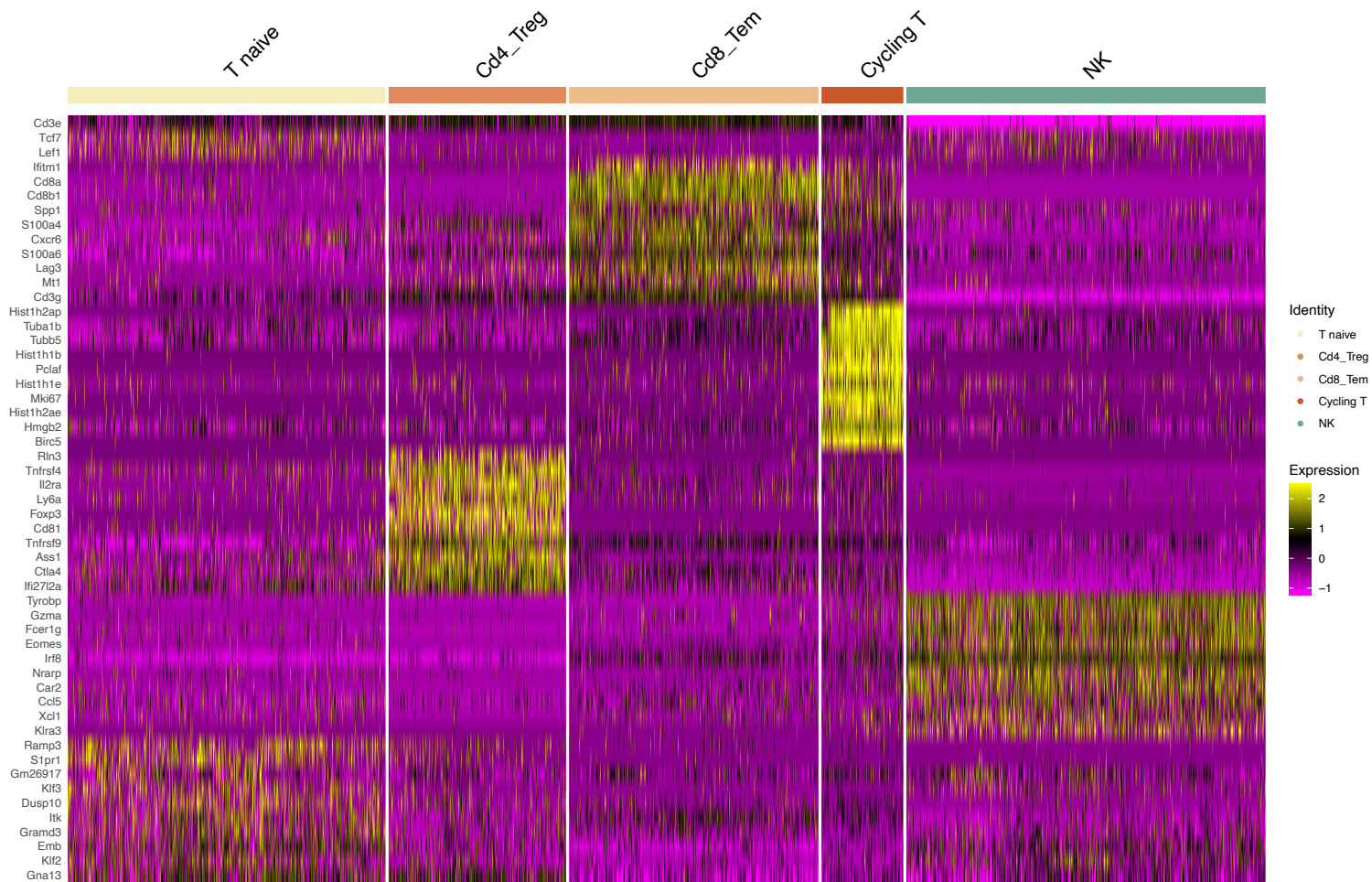

Supplement: Supplementary file 1 [file antioxidants-13-00477-s001.zip › Figure S4.pdf]

**A**

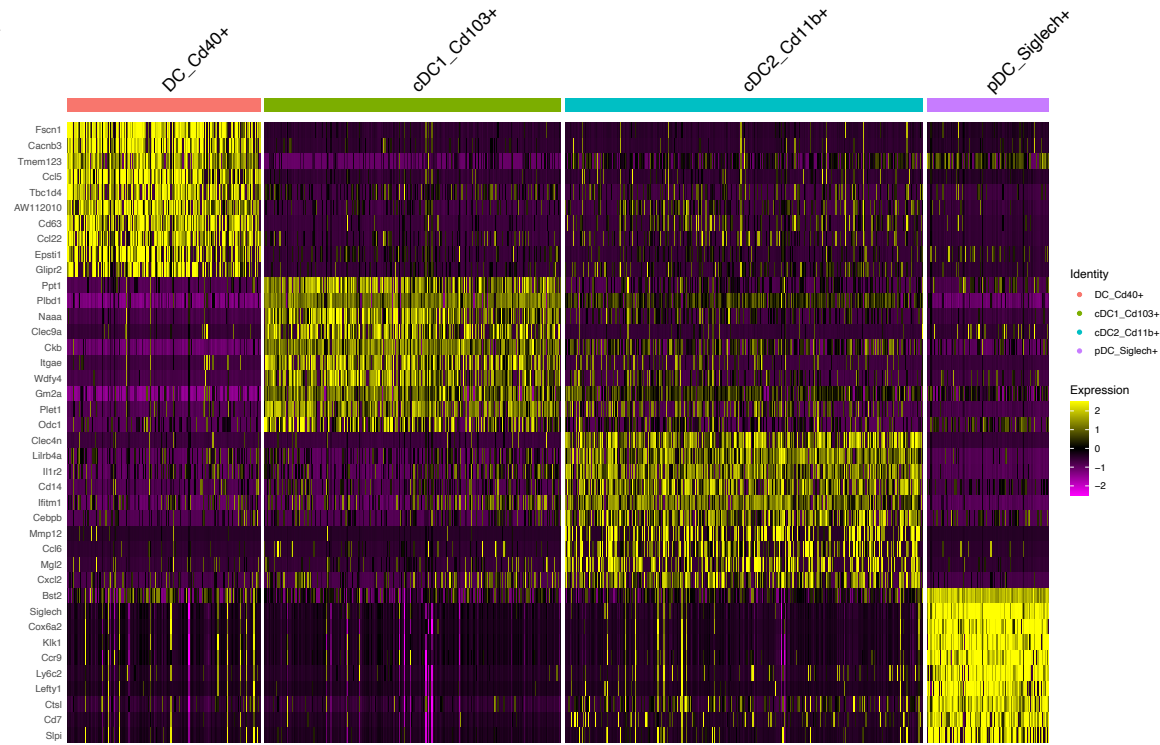

**B**

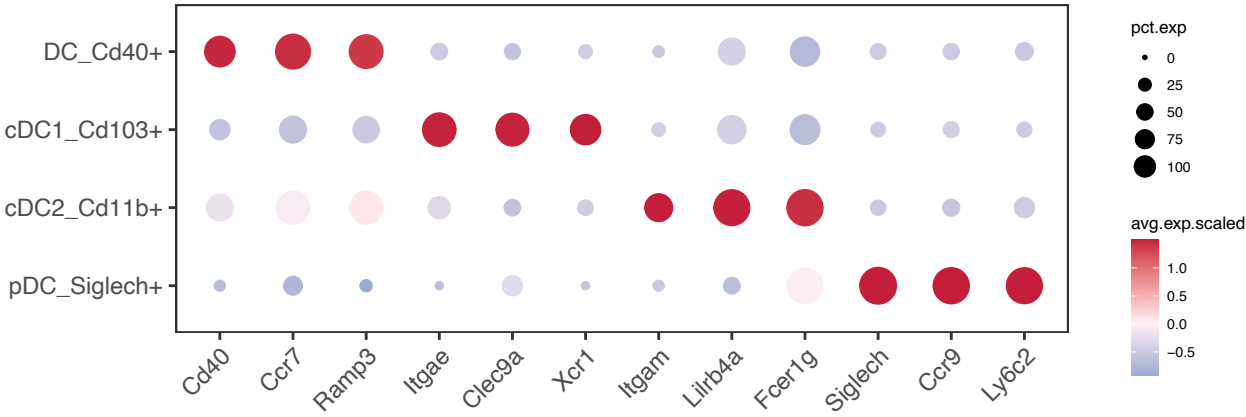

Supplement: Supplementary file 1 [file antioxidants-13-00477-s001.zip › Figure S5.pdf]

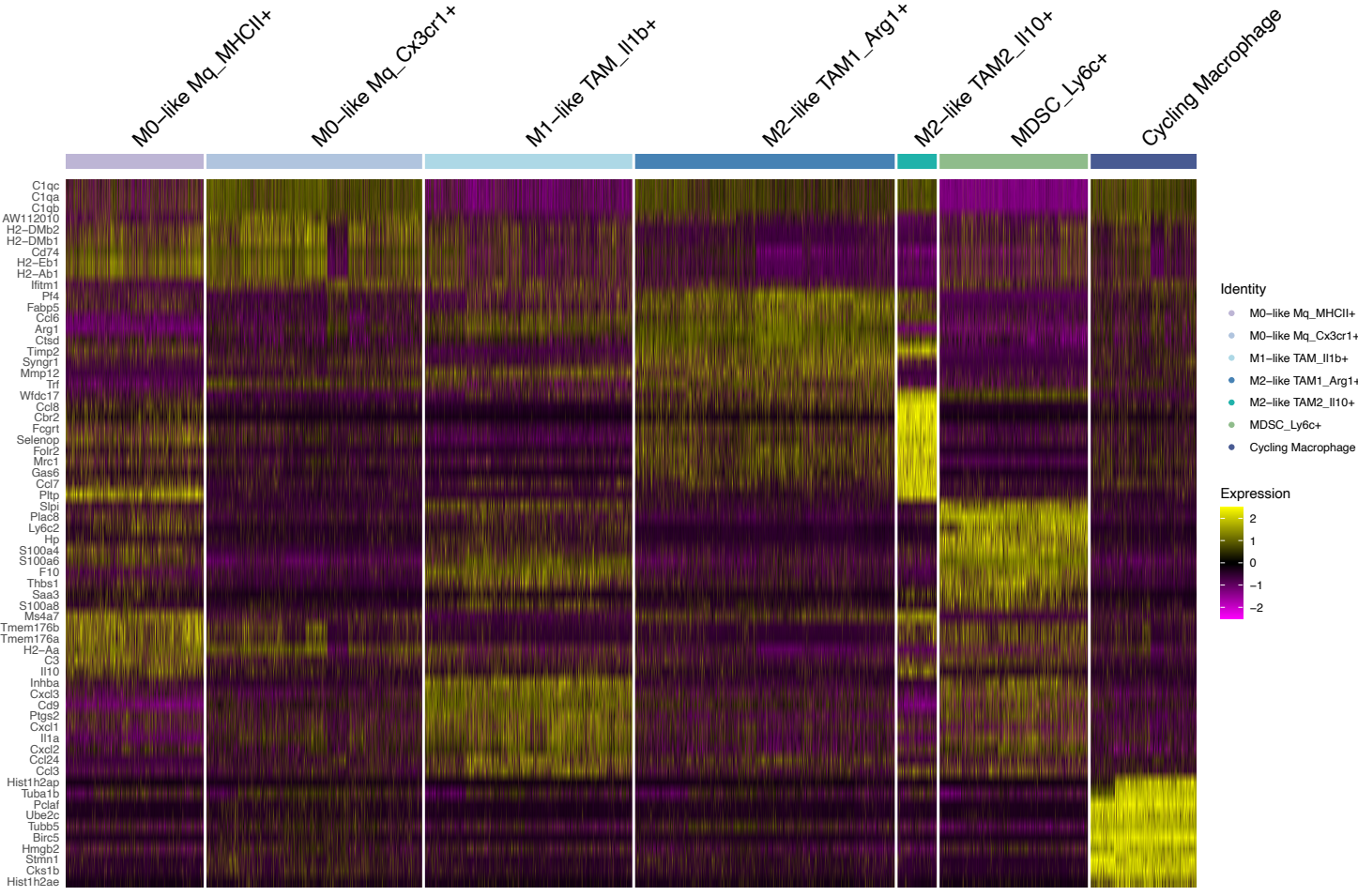

Supplement: Supplementary file 1 [file antioxidants-13-00477-s001.zip › Figure S6.pdf]

**A**

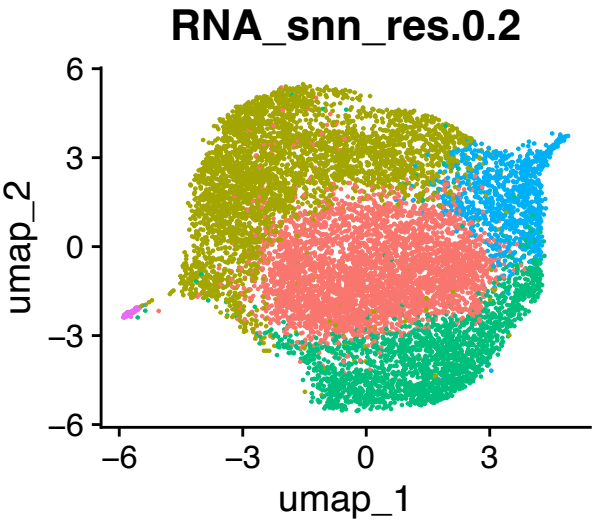

**B**

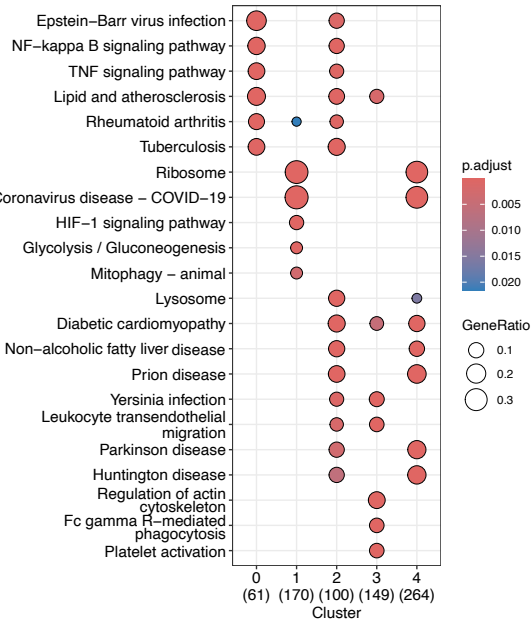

**C**

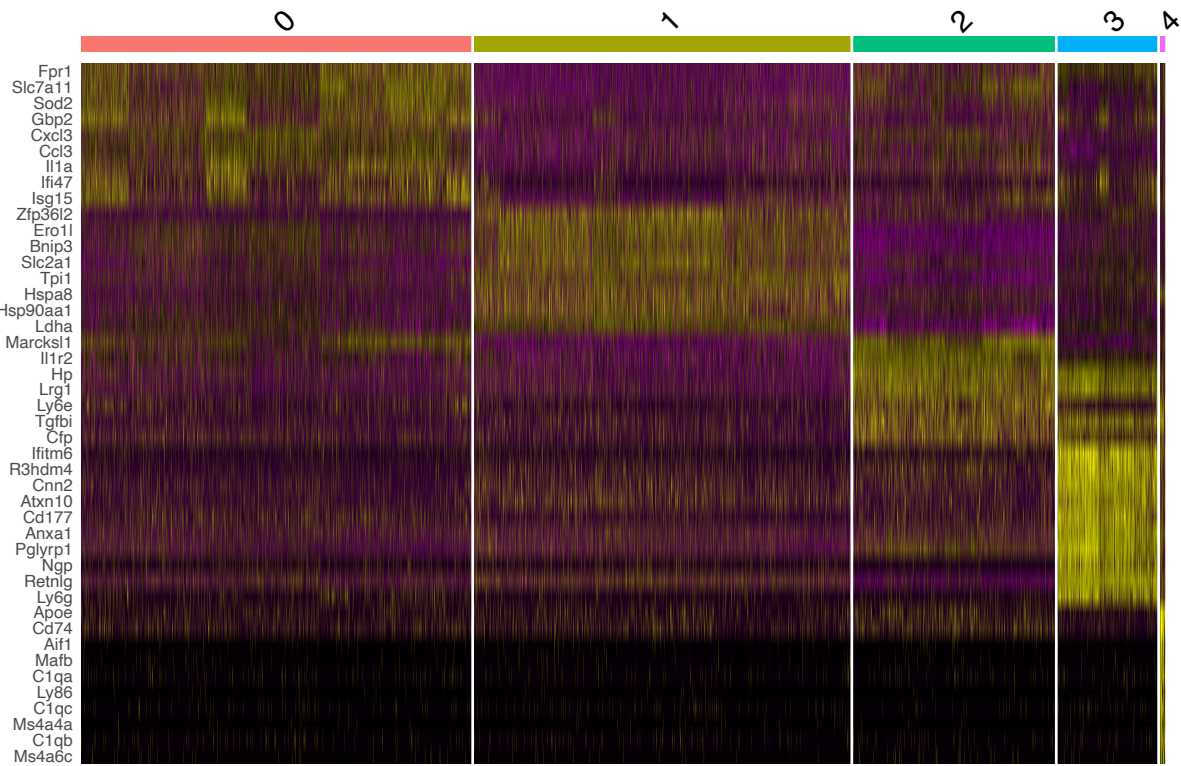

Supplement: Supplementary file 1 [file antioxidants-13-00477-s001.zip › Figure S7.pdf]

**A**

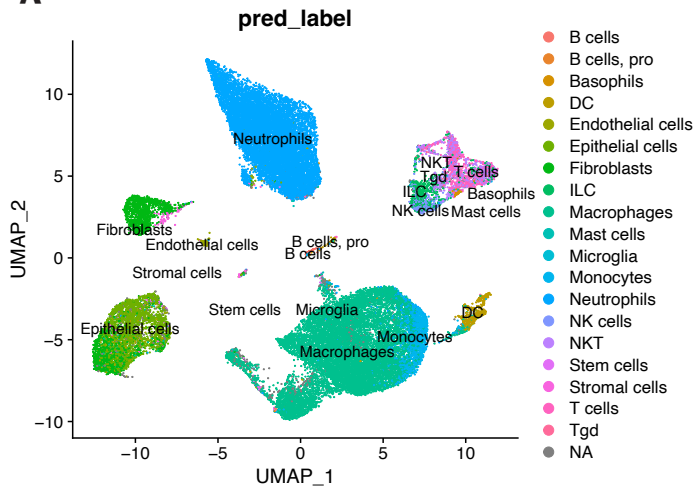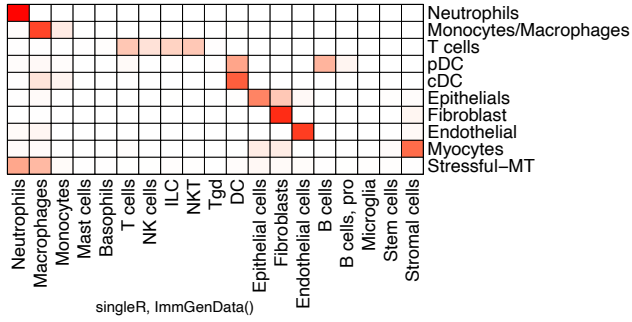

**B**

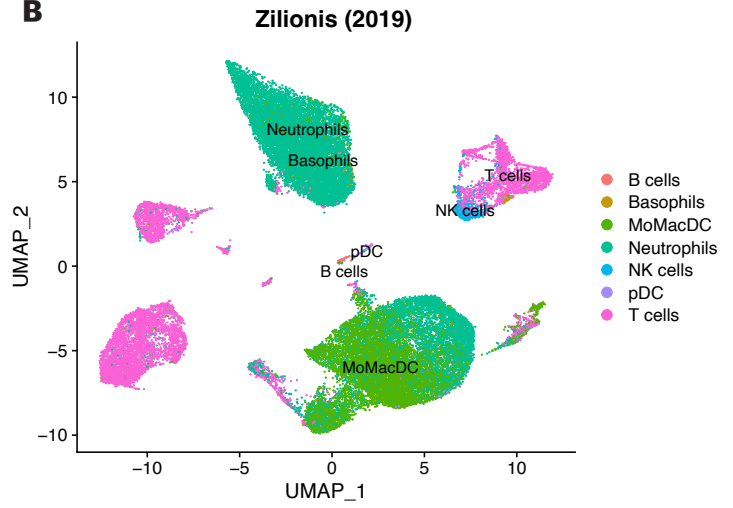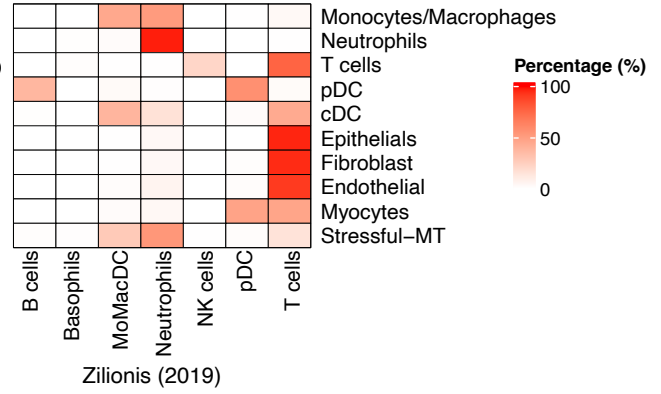

**C**

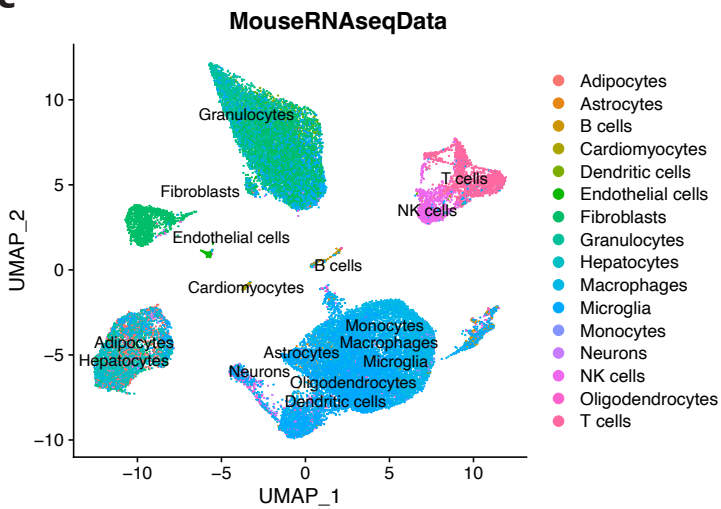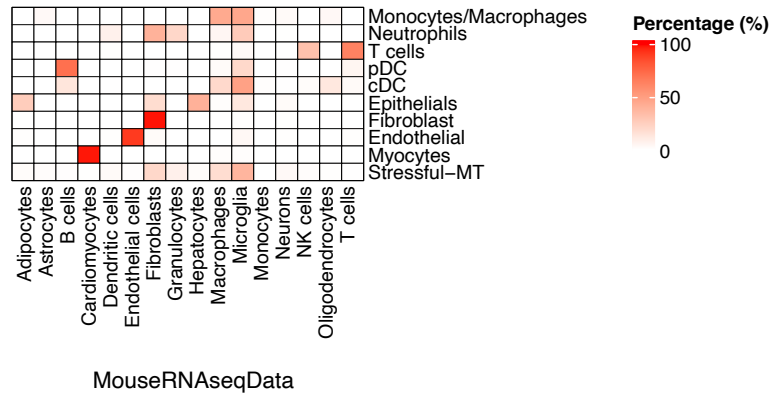

Supplement: Supplementary file 1 [file antioxidants-13-00477-s001.zip › Figure S8.pdf]
